# A soft supernumerary hand for rehabilitation in sub-acute stroke: a pilot study

**DOI:** 10.1038/s41598-022-25029-0

**Published:** 2022-12-13

**Authors:** Carlo Trompetto, Manuel G. Catalano, Alessandro Farina, Giorgio Grioli, Laura Mori, Andrea Ciullo, Matteo Pittaluga, Martina Rossero, Luca Puce, Antonio Bicchi

**Affiliations:** 1 Department of Neuroscience, Rehabilitation, Ophthalmology, Genetics, Maternal and Child Health, University of Genova, 16132 Genova, Italy; 2 Neurorehabilitation Unit, Department of Neuroscience, IRCCS Ospedale Policlinico San Martino, Genova, 16132 Genova, Italy; 3 Soft Robotics for Human Cooperation, and Rehabilitation Lab, Fondazione Istituto Italiano di Tecnologia, 16163 Genova, Italy; 4 Centro di Ricerca “Enrico Piaggio” and Dipartimento di Ingegneria dell’Informazione, Università di Pisa, 56122 Pisa, Italy

**Keywords:** Rehabilitation, Stroke

## Abstract

In patients with subacute stroke, task specific training (TST) has been shown to accelerate functional recovery of the upper limb. However, many patients do not have sufficient active extension of the fingers to perform this treatment. In these patients, here we propose a new rehabilitation technique in which TST is performed through a soft robotic hand (SoftHand-X). In short, the extension of the robotic fingers is controlled by the patient through his residual, albeit minimal, active extension of the fingers or wrist, while the patient was required to relax the muscles to achieve full flexion of the robotic fingers. TST with SoftHand-X was attempted in 27 subacute stroke patients unable to perform TST due to insufficient active extension of the fingers. Four patients (14.8%) were able to perform the proposed treatment (10 daily sessions of 60 min each). They reported an excellent level of participation. After the treatment, both clinical score of spasticity and its electromyographic correlate (stretch reflex) decreased. In subacute stroke patients, TST using SoftHand-X is a well-accepted treatment, resulting in a decrease of spasticity. At present, it can be applied only in a small proportion of the patients who cannot perform conventional TST, though extensions are possible.

## Introduction

Stroke is the leading cause of disability in western society^1^. Six months after stroke, approximately 50% of patients remain with a chronic reduction of arm function^2^. That reduces their independence and quality of life significantly^3^. Therefore, improving upper limb function is a core element of stroke rehabilitation.

It is widely accepted that, to promote neural plasticity and functional improvement, upper limb motor training should draw inspiration from the principles of motor learning. According to these principles, a technique known as task-specific training (TST) has been developed. TST is based on repetitive and intensive goal-directed motor tasks, which are meaningful to the patient and whose difficulty levels are progressively adapted to his/her abilities^4^. In stroke patients, TST has been shown to accelerate functional recovery of the upper limb^5^.

The natural course of clinical recovery after stroke reflects the ability of the brain to adapt plastically to injury^6^. Observational studies show that recovery is more rapid during the first month after stroke, and motor function typically reaches a plateau within 3 months^7^. Compelling evidence state that the first three months after stroke (sub-acute phase) may represent a critical window for rehabilitation to maximize recovery of body functions and activities^8,9^.

Unfortunately, the sub-acute phase is also the period in which spasticity appears. At its onset, which usually happens in the first few weeks after stroke, spasticity does not cause disability. While the prevalence of spasticity peaks at four weeks after stroke, the number of patients with severe spasticity (spastic dystonia) continues to increase during the first year^10^. At one year after stroke, spastic dystonia of fingers, wrist, and elbow flexors can be very disabling, requiring chronic treatment with botulinum toxin^11^.

In the sub-acute phase, several stroke patients do not have active finger extension. Although many of them retain the ability to voluntarily activate finger flexors, spasticity often appears in these muscles and there is the concern, deeply felt by physiotherapists, that repetitive and intense flexion movements may turn spasticity into spastic dystonia^12^. The incapability of voluntarily activating finger extensors and the concern to worsen spasticity through the voluntary activation of finger flexors make TST a difficult approach. Because of this, physiotherapists usually focus their efforts with these patients on the trunk and the proximal segment of the upper limb, with the aim to improve postural control. It has been shown that such patients with no active finger extension tend to present a poor motor recovery of the upper limb in the following months^13^.

Robotics is a promising approach to post-stroke rehabilitation. It may be used to deliver or enhance TST^14^. Robots for upper limb training differentiate into exoskeletons and end-effector robots. While exoskeletons control one or more joints of the paretic limb by means of torque actuators, end-effector robots guide only the most distal part of the paretic limb. A recent meta-analysis states that, in comparison with non-robotic treatment, robotic rehabilitation of the upper limb produces a positive effect on motor control and muscle strength of the paretic limb, but a negative effect on spasticity^15^.

Supernumerary Robotic Limbs (SRLs) represent a third, novel category of rehabilitation robotics, which can be considered as wearable robots augmenting the human body by providing additional robotic limbs or fingers. Unlike exoskeletons, SRLs do not require joint-to-joint alignment. Moreover, they do not force the user to follow a specific trajectory with their own body parts. SRLs have been initially proposed for industrial purposes to improve users' ergonomic comfort and enhance their capabilities^16,17^. Recently, SRLs have been used in chronic stroke patients for compensating their missing abilities and counter learned non-use^18,19^. To date, SRLs have never been used in post-acute stroke patients to promote neural plasticity and functional improvement.

**Figure 1:**
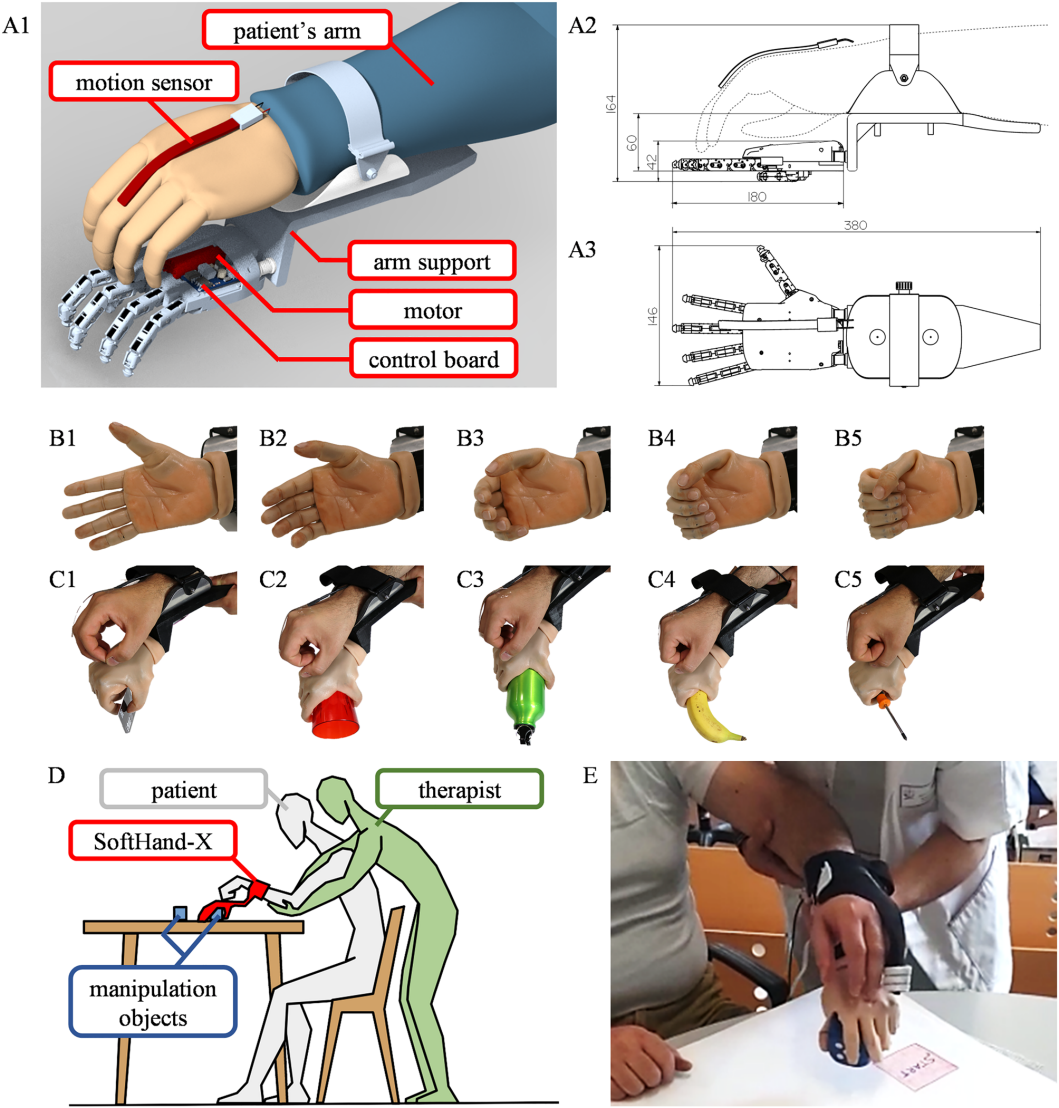
Rehabilitation setup. (**A1**) SoftHand-X Supernumerary Robotic Hand and its main subsystems, including the hand hardware and the sensing system used to convert motions of the patient into motions of the robot, top view (**A2**) and side view (**A3**), with main system dimensions (in mm). (**B**) Photographic sequence (**B1**, **B2**, **B3**, **B4**, **B5**) of the motion pattern of the supernumerary robotic hand, inspired to the most statistically frequent human hand motion, described in literature as the first postural synergy of grasping. (**C**) Grip patterns of the soft supernumerary robotic hand conforming to the shape of different objects to achieve a natural-looking grasp posture: (**C1**) a credit card, (**C2**) a drinking glass, (**C3**) a bottle, (**C4**) a banana, and (**C5**) a screwdriver. (**D**) Illustration of the full rehabilitation scenario: a patient (white) is sat comfortably and wears the supernumerary limb (red); a therapist (green) guides the patient in the manipulation of objects (blue) resting on a table. (**E**) Close-up picture of one of the rehabilitation phases: the patient must reach a plastic cube placed on the table, grab it, and then release it. The physiotherapist supports the arm and forearm, facilitating the patient's proximal movements. The grip and release movements are performed through the robotic hand under patient’s control, without the help of the physiotherapist.

In this pilot study, a supernumerary robotic hand (SoftHand-X) was used to administer TST in subacute stroke patients with insufficient active finger extension to perform meaningful, goal-directed motor tasks (see Fig. 1). In these patients, hand rehabilitation is usually limited to mirror therapy^20^ and passive movements. In our protocol, extension of the robotic fingers is controlled by the patient through his/her residual, albeit minimal, active extension of fingers or wrist, while the patient was required to relax their muscles to achieve full flexion of the robotic fingers. Our idea is that, following this protocol, patients could be able to perform TST without any overt activation of flexor muscles of the wrist and fingers, whose intensive and repetitive activation could favor the development of spastic dystonia in the following months.

The primary aim of this pilot study is twofold: (a) among patients with subacute stroke who cannot be treated with conventional TST (i.e., without the use of SoftHand-X) due to finger extensor muscle weakness, find the percentage of patients who could be treated with TST using SoftHand-X guided by residual finger or wrist extension movements (henceforward called “suitable patients”); (b) assess the compliance of the “suitable patients” to the proposed treatment (i.e., TST using SoftHand-X guided by residual finger or wrist extension movements) using the Pittsburgh Rehabilitation Participation Scale (PRPS)^21^.

The secondary aim is the follow-up of muscle tone in “suitable patients” treated with SoftHand-X, up to one year after the acute event, using the modified Ashworth scale (MAS) and stretch reflex assessment.

## Results

From 1 April to 30 September 2020, 27 sub-acute stroke patients met the inclusion criteria (i.e., resulted unable to grasp and release objects due to insufficient active finger extension) and were enrolled in the study.

Among these 27 patients, 23 patients (85.2%) were not able to perform TST using the robotic hand (age 67.00 ± 9.72 years; 9 women) and were used as control group. Fifteen patients (55.6%) had no finger or wrist extension movement. Six patients (22.2%) had some small active extensions of the wrist and/or fingers; however, these movements resulted too small (< 5°) to be reliably distinguished from tremor and sensor noise. Two patients (7.4%) were able to control the robotic hand by extending the wrist, but after a few movements the range of motion covered by the active movements was reduced due to fatigue and the patients could no longer control the robotic hand.

Among the 27 patients who met the inclusion criteria, four patients (14.8%) were able to control the robotic hand (“suitable patients”) and underwent TST using SoftHand-X (experimental group) (Table 1). Three of them used the index finger to guide the robotic hand (proximal interphalangeal joint), while patient 2 used the wrist in the first four sessions and the index finger (proximal interphalangeal joint) in the remaining six. None of the patients experienced any adverse effect during the 10 treatment sessions (5 days a week for 2 weeks). All the patients reported excellent participation in PRPS in most of the 10 sessions (patient 2: excellent participation at each session; patient 1 and 4: excellent participation in correspondence to 8/10 sessions; patient 3: excellent participation in correspondence to 7/10 sessions). The minimum and maximum values reported by the patients were respectively 4 and 6 and the first quartile was 5 for patient 3 and 6 for the remaining three patients.

**Table 1:** Demographic and clinical characteristics of patients.

	Age (years)	Sex	Stroke type	Paretic side	Time between stroke onset and T0 (days)
Patient 1	57	Female	Haemorrhagic	Left	50
Patient 2	68	Male	Ischaemic	Left	45
Patient 3	74	Male	Ischaemic	Left	18
Patient 4	58	Male	Ischaemic	Left	60

**Figure 2:**
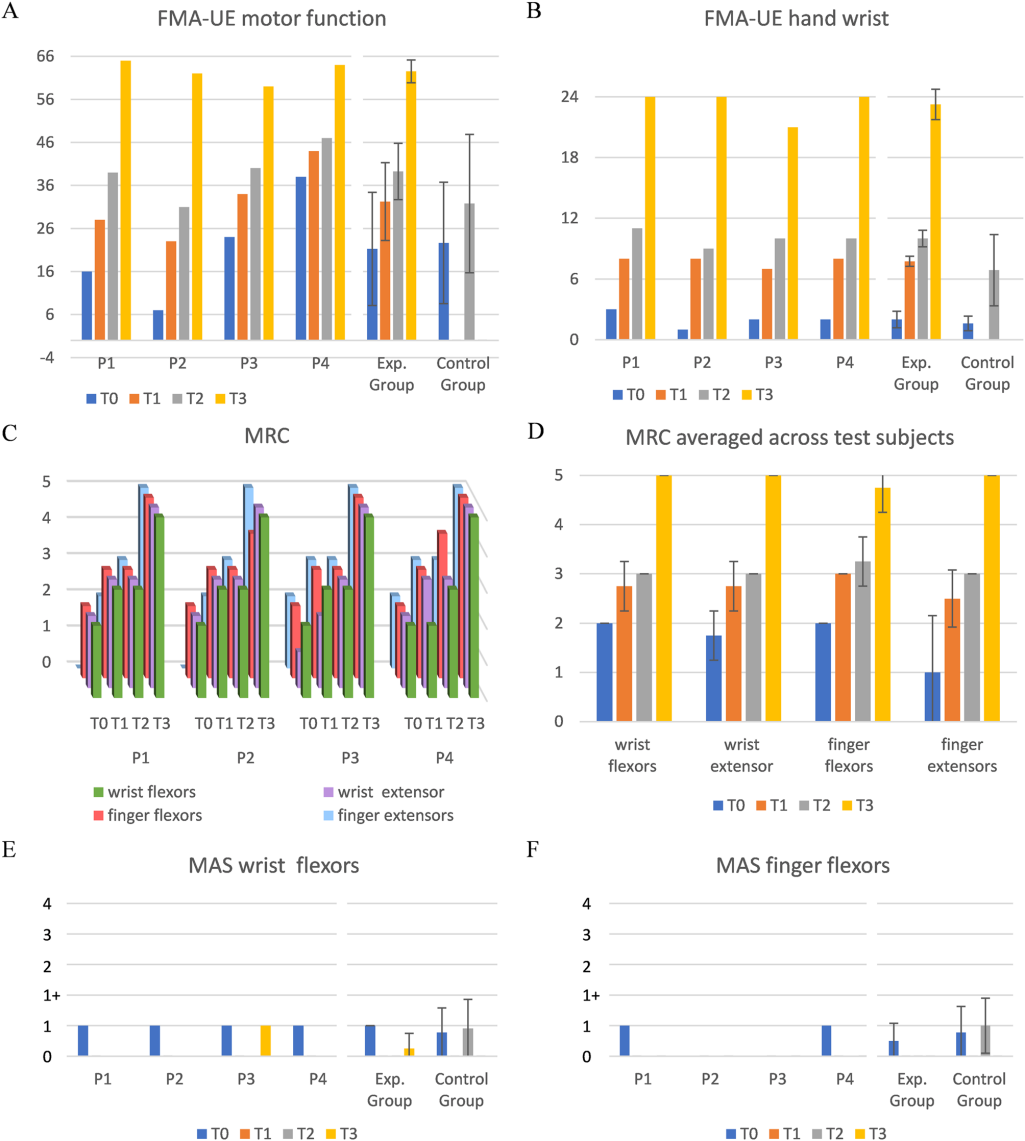
Clinical scores in experimental and control group. (**A**) Motor section of Fugl–Meyer Assessment for Upper Extremity (FMA-UE) and (**B**) hand-wrist domain of FMA-UE motor section; experimental group patients P1 to P4 (left), mean and standard deviation of experimental and control group (right). The motor section of FMA-UE is scored out of 66, with sub-scores of 24 for the wrist and hand. (**C**) Medical Research Council (MRC) for the strength of wrist and finger flexor and extensor muscles for each patient of the experimental group, and (**D**) means and standard deviation across patients of the experimental group. MRC is rated from 0 = no contraction, to 5 = normal strength. (**E**) MAS for the wrist flexors and (**F**) for the finger flexors; patients P1 to P4 (left), mean and standard deviation across experimental and control group (right). The MAS rates muscle tone from 0 (no increased muscle tone) to 4 (rigid flexion or extension). Note that control group data for panels (**A**), (**B**), (**E**) and (**F**) is available only at T0 and T2.

Figure 2 shows the clinical scores of the four patients of the experimental group evaluated at the 4 time points: at the enrolment in the study (before the treatment) (T0), just after the last session of treatment (T1), 25 days after T0 (T2) and 1 year after stroke (T3). The Fugl–Meyer Assessment for upper extremity (FMA-UE)^22^ was 21.25 ± 13.15 at T0, 32.25 ± 9.03 at T1, 39.25 ± 6.55 at T2, and 62.5 ± 2.65 at T3 (Fig. 2A). The hand/wrist motor sub-score of FMA-UE was 2.00 ± 0.82 at T0, 7.75 ± 0.50 at T1, 10.00 ± 0.82 at T2, and 23.25 ± 1.50 at T3 (Fig. 2B). Moreover, the Medical Research Council (MRC) scores increased in all patients, indicating a progressive improvement (from T0 to T3) of strength in flexor–extensor muscles of the wrist and fingers (Fig. 2C,D). At T0, two patients (1 and 4) showed an MAS score of 1 (mild spasticity) in flexor muscles of both wrist (Fig. 2E) and fingers (Fig. 2F), while the other 2 patients showed an MAS of 1 only in wrist flexors. A decrease of the MAS score from 1 to 0 (no spasticity) was observed at T1 and at T2 in all 4 patients. At T3, a mild spasticity in wrist flexors (MAS 1) reappeared only in patient 3 (Fig. 2E).

In patients of the control group, FMA-UE score was 22.65 ± 14.10 at T0 (at the enrolment in the study), and 31.78 ± 16.07 at T2 (25 days after T0) (Fig. 2A), while hand/wrist motor sub-score was 1.61 ± 0.72 at T0, and 6.87 ± 3.52 at T2 (Fig. 2B). Wrist MAS score was 0.78 ± 0.80 at T0, and 0.91 ± 0.95 at T2 (Fig. 2E), while fingers MAS score was 0.78 ± 0.85 at T0, and 1.00 ± 0.90 at T2 (Fig. 2F).

No significant differences between experimental and control group were found at T0 for both FMA-UE score and MAS score (FMA-UE total score: U = 44, p = 0.9; FMA-UE hand/wrist sub-score: U = 32.5, p = 0.3; wrist MAS score: U = 36, p = 0.5; fingers MAS score: U = 39, p = 0.6). At T2, the two groups scored similarly for FMA-UE total score (U = 29.5, p = 0.3) and hand/wrist sub-score (U = 23, p = 0.1), while fingers MAS score was significantly lower in the experimental group (U = 16, p = 0.029); for wrist MAS score only a trend for lower score in the experimental group emerged (U = 20, p = 0.053).

**Figure 3:**
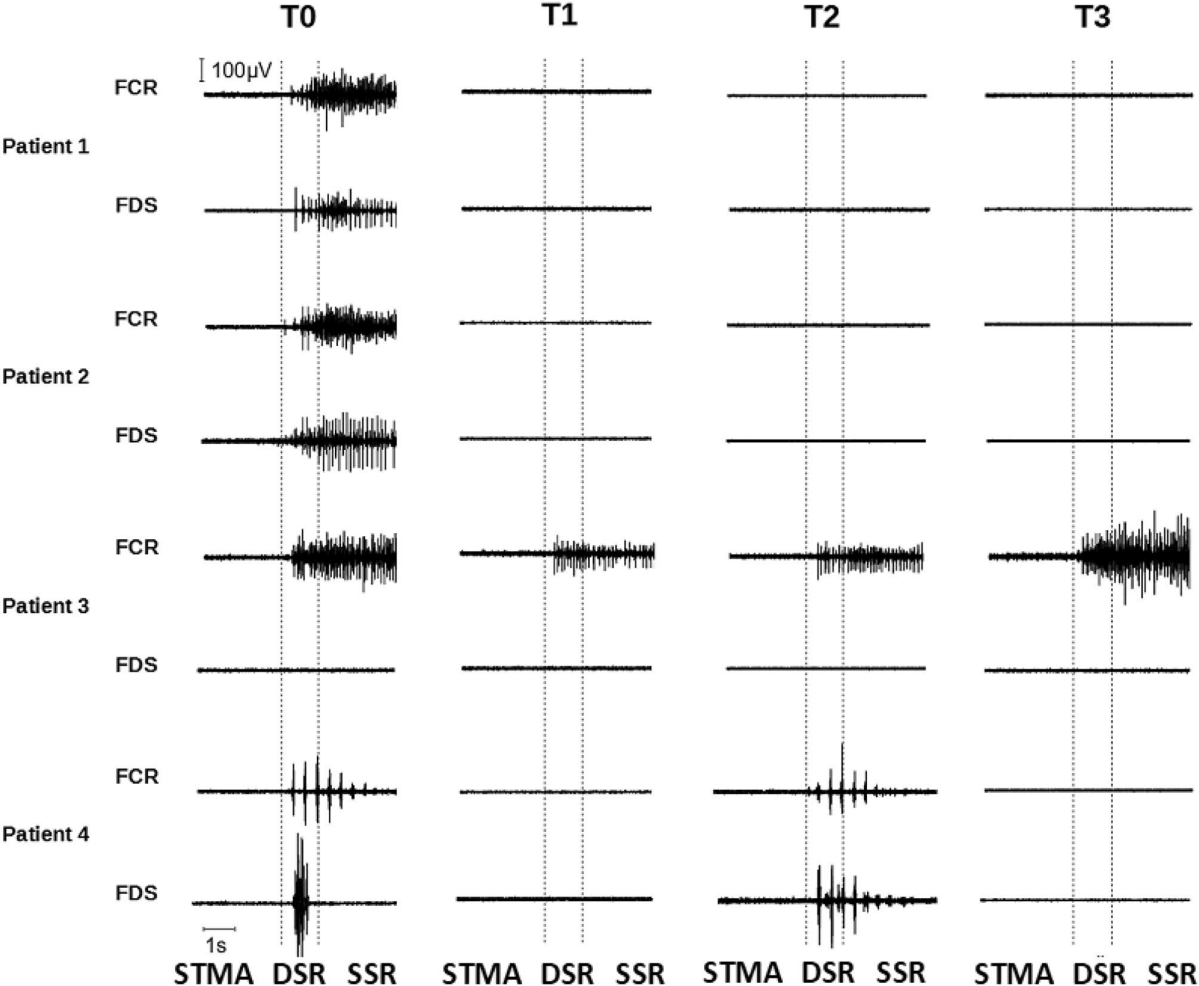
EMG activity of Flexor Carpi Radialis (FCR) and Flexor Digitorum Superficialis (FDS) produced by passive stretching in the four patients of the experimental group. First vertical dotted line indicates the start of passive stretch of the wrist and fingers, while the second vertical dotted line indicates the end of passive stretch. Spontaneous Tonic Muscle Activity (STMA) is before the first line, Dynamic Stretch Reflex (DSR) is between the two lines, and Static Stretch Reflex (SSR) is after the second line (see “Stretch reflex assessment and measurement”). No EMG activity was detected prior to passive stretch in any patient (i.e., STMA was not found in any patient). Spasticity (i.e., DSR with or without SSR) was present in all 4 patients at T0 in both FCR and FDS, with the exception of patient 3, in whom spasticity was present only in FCR. After treatment (T1), spasticity disappears in patients 1, 2, 4 and decreases in patient 3.

In the experimental group, EMG assessment of muscle tone (Fig. 3) showed, at T0, a stretch reflex not preceded by a tonic muscle contraction (i.e., spasticity) in all 4 patients. In the first 2 patients, spasticity disappeared immediately after treatment (T1) and never returned (T2 and T3). In patient number 3, spasticity decreased after treatment (T1 and T2), while at the last evaluation (T3) it was slightly more intense than at baseline. Finally, in the last patient, spasticity disappeared immediately after treatment (T1), it reappeared 10 days after the end of treatment (T2), while it was not present at the last assessment (T3).

## Discussion

Primary outcome: (a) find the “suitable patients”.

We enrolled 27 sub-acute stroke patients in whom TST of the upper limb was no possible due to deficit of active finger extension. In these patients, unable to perform any goal-directed motor tasks with their affected hand, SoftHand-X represents a new possibility to perform TST.

Within this population of 27 patients, only four patients (14.8%) were able to perform TST with SoftHandX ("suitable patients"). Our current protocol requires that patients be able to perform detectable wrist or finger extension movements, even if minimal. The results of this pilot study show that the requirement of such movements is the main limiting factor for a wider applicability.

Primary outcome: (b) assess the compliance of the “suitable patients” to TST with SoftHand-X.

According to the inclusion criteria, the four “suitable patients” who made up the experimental group were unable to grasp and release objects. Instead, using SoftHand-X, they became able to perform these tasks and could follow an intensive rehabilitation program focused on grasping and releasing objects of various shapes and sizes. None of the participants experienced any adverse effect during the 10 treatment sessions (5 days a week for 2 weeks). Patients’ compliance was assessed measuring the level participation to the treatment, by means of the PRPS. For each patient, the PRPS mode among the 10 evaluations was 6, meaning “excellent participation in all exercises with maximal efforts, finishing all exercises, and taking an active interest in exercises and/or future therapy sessions”. Interestingly, patient 1 and patient 3 reported values lower than 6 only in their first assessments and then reported excellent participation consistently in all the remaining evaluations.

Secondary aim: follow-up of muscle tone in patients of the experimental group, up to one year after the acute event, using the modified Ashworth scale (MAS) and stretch reflex assessment.

The four patients of the experimental group, before starting the treatment (T0), had a severe functional impairment of the wrist and hand (Fig. 2A,B). They also had a slight hypertonia of the wrist flexor muscles (Fig. 2E) and, limited to 2 of them (patients 1 and 4), also of the fingers (Fig. 2F). Statistical analysis did not find any difference at baseline (T0) between experimental and control group for FMA-UE scores (both total and wrist/hand sub-score) and MAS score. In the four patients of the experimental group, EMG recording showed a stretch reflex in wrist and/or finger flexors without spontaneous tonic muscle activation prior to passive muscle stretch (Fig. 3). This finding documents the presence of spasticity, i.e. exaggeration of the stretch reflex, which can be evoked at the low rates of passive stretch used to assess clinically the muscle tone^23^.

Such association between spasticity and severe motor impairment in the first 3 months after stroke is an important predictor of the possible development of spastic dystonia one year after stroke^11^. While spasticity is the exaggeration of the stretch reflex^24^, spastic dystonia is the inability to relax the muscles voluntarily, characterized by a spontaneous tonic muscle activation when the subject tries to relax. Although spastic dystonia is present at rest, without any muscle stretch, it is stretch-sensitive, meaning that it increases when the muscle is stretched, leading to muscle hypertonia, as occurring in spasticity^25^. Spastic dystonia is very disabling, impairs upper limb function, and causes pain and secondary muscle changes^23^.

In the control group, from T0 to T2, mean FMA-UE score increased by 9 points and hand-wrist sub-score increased by 5 points. Although this increase was lower than that observed in the four patients of the experimental group (18 points for FMA-UE score and 8 points for hand-wrist sub-score), at T2 no significant between-group differences were found. This is a widely expected result, given the low sample number and large variability of the data.

On the other hand, while in the control group MAS score remained substantially stable, in each one of the four subjects of the experimental group MAS score decreased from T0 to T2. Significant between-group differences were found at T2, with a lower MAS score for fingers and a trend for a lower MAS score for wrist in the experimental group.

Therefore, the data from this pilot study suggest that, in sub-acute stroke patients unable to perform TST, the proposed treatment (TST with SoftHand-X) can reduce spasticity.

Finally, it is important to note that one year after the stroke, when spastic dystonia tends to reach its peak^11^, none of the 4 patients showed spastic dystonia.

From a theoretical point of view, performing TST with SoftHand-X could promote functional recovery of the hand through various mechanisms. First, when performing TST with SoftHand-X, patients have the illusion that the robotic hand is their hand, as reported by each of the four patients in the experimental group. This illusion could activate the mirror neuron system and promote motor recovery, with a similar mechanism to that postulated for mirror therapy, action observation, and motor imagery^26^, all methods extensively used in stroke patient rehabilitation. Second, while performing TST with SoftHand-X, patients repeatedly and intensively activate extensor muscles of the wrist and fingers, promoting functional recovery of extensor muscles and their inhibitory control on flexor ones (reciprocal inhibition). In stroke patients, reduced reciprocal inhibition from extensors to flexors is a well-recognized cause of upper limb spasticity^27^. Finally, performing TST with SoftHand-X does not require repeated and intense voluntary activation of flexor muscles of the fingers, i.e. those in which spasticity and spastic dystonia tend to appear. To flex the robotic fingers, patients are asked to relax their extensor muscles. In stroke patients, repetitive and intense voluntary activation of flexor muscles could favor the development of spasticity and spastic dystonia, since spinal motoneurons are excited by para-pyramidal tracts (e.g., the ventral reticulo-spinal tract), which are known to have a positive effect on muscle tone^23^.

Although the overwhelming majority of TST evidence relates to stroke, TST has been proved effective also in other diseases, such as traumatic brain injury, parkinsonism, and spinal cord injury^28^. Therefore, TST with SoftHand-X could be exploited also in these conditions.

Besides rehabilitative approaches, a robotic hand to substitute a damaged limb has previously been proposed in patients with severe hand disabilities, including global plexopathies^29^ and critical soft tissue injuries^30^. SoftHand-X has also been shown to compensate for severely impaired hand function in chronic stroke patients^19^. These compensatory approaches can be extremely helpful in patients with chronic conditions. However, in the subacute phase of stroke, they could hinder the plastic reorganization of the brain, thus limiting functional recovery.

### Limitations

Although clinical scores at baseline (FMA-UE and MAS) did not differ between experimental and control group, it must be emphasized that the patients were not randomly assigned to the two groups. The control group was assigned patients who were unable to use the robotic hand to perform TST, while the experimental group was assigned patients able to do so. Therefore, it is not possible to exclude that this difference may have played a role in determining the functional outcomes in the two groups.

Another limitation of our study consists in the small sample size of the experimental group. Specifically, due to the nature of our mono-centric pilot study, only four “suitable patients” were found, and thus these results are to be considered as preliminary. Future research should surely enlarge the sample, maybe including more centers, considering a larger enrollment period, and using alternative signals to control the robotic hand. A larger sample of “suitable patients” could enable to make randomization into the two groups.

## Conclusions

In sub-acute stroke patients, with severe hand impairment that prevents them from performing goal-directed tasks, TST using SoftHand-X is a well-accepted treatment, resulting in a decrease of spasticity.

Using the small residual extension movements of the fingers and wrist to control the robotic hand, only 14.8% of patients not eligible for standard TST could benefit from the treatment.

Preliminary data (not reported here) with the use of alternative signals to control SoftHand-X (e.g., through EMG measurements from extensor muscles of the wrist and fingers) suggest that these modalities could significantly increase the proportion of “suitable patients”.

## Methods

### Participants

Eligible patients were sought from sub-acute stroke patients admitted to our intensive neuro-rehabilitation unit from 1 April to 30 September 2020, who met the following criteria:

- first-ever stroke, occurred no longer than 2 months before the admission,
- ischemic or hemorrhagic lesions confirmed by computed tomography or magnetic resonance imaging,
- upper limb functional impairment with impossibility to grasp and release objects due to finger extension deficit.

The present study was carried out in accordance with the Code of Ethics of the World Medical Association (Declaration of Helsinki) for experiments involving humans; a written informed consent was obtained from all participants. The study was approved by the local ethics committee of “IRCCS-Ospedale Policlinico San Martino, Genova, Italia” (approval number 258REG2017). Demographic and clinical data of the subjects is reported in Table 1.

### Robotic hand

The SoftHand-X (SoftHand eXtrathesis) is a derivation of the robotic hand prosthesis SoftHand Pro^31^. The hand has 19 independent joints that approximate well the position and range of the main articulations of the human hand. The hand design and control are based on the concept of soft synergies^32^, which in turn draws from the analysis of human motor control organization in synergies^33^ and from the equilibrium point hypothesis^34^. The implementation of this concept through a morphological intelligence approach to the hardware and firmware design makes the overall control of the hand extremely simple: a single open/close input from the patient can control all the 19 independent joints. The hand is capable of morphing itself around objects of different shapes and sizes, grasping and manipulating them in different functional ways. Soft robotics techniques and materials employed in the construction of the hand also make the fingers resilient to impacts and joint dislocations and contribute to the feeling of compliance and naturalness in physical social and self-interaction^35^. These characteristics, together with a carefully manufactured cosmetic glove, make for a high degree of anthropomorphism of the hand both aesthetical and functional.

Overall dimensions of the SoftHand-X are: 20 cm (palm + middle finger length), 10 cm (palm width) and 5 cm (palm thickness). Maximum grasping forces are: 80 N power grasp (cylindrical object, diameter 80 mm), 30 N pinch grasp (flat object, height 2 mm). Weight: around 500 g. The SoftHand-X is powered by a Maxon Motor DCX22S equipped with a GPX22 planetary gearbox. An Austrian Microsystem magnetic encoder is used to read the position of the motor and close the control loop. A custom electronic board drives the hand and interfaces with input devices. Many different types of input devices can be used to detect the patient’s grasp intention and drive the hand, ranging from EMGs in different locations to force handles, bending sensors, and triggers^19^.

### Input interface

To control the opening and closing of Soft-Hand X, in this pilot study we used an input method based on the measurement of the extension of the fingers or the wrist of the affected side through a bending sensor (resistive flex Sensor, Spectra Symbol). The detection of an extension movement of the patient above a subject-specific threshold controlled the opening of the robotic fingers, while the relaxation of the extensors determined the closing of the robotic fingers. In the search for "suitable patients", the sensor was simply placed where the largest and most reproducible movements were present. We first tried using finger extension movements to guide the robotic hand. The cursor was positioned on a finger (excluding the thumb), at the proximal interphalangeal joint or the metacarpophalangeal joint. In case of inability to guide the robotic hand due to lack of usable movements of the selected finger, we tried to use the wrist extension movement and the sensor was placed on the wrist.

### Treatment with the robotic hand and conventional treatment

Patients who met the inclusion criteria and were able to perform TST using SoftHand-X (“suitable patients”) formed the experimental group. Patients who met the inclusion criteria but were unable to perform TST using SoftHand-X formed the control group.

During the treatment with the robotic hand, experimental group patients were sitting on a chair in front of a table. The treatment was based on a few specific tasks (ranging from 2 to 4, according to subject’s compliance and capacity), which were intensively practiced for several repetitions. The tasks called for reaching, grasping and manipulating objects; examples include putting pegs into holes, stacking cones and checkers. The proximal part of the upper limb (the arm and, if necessary, also the forearm) was supported by the physiotherapist, who helped the patient in the postural part of the task, made difficult not only by the lack of strength, but also by the weight of the robotic hand. The part of the task involving grasping and manipulating objects was instead performed entirely by the patient through the robotic hand (Fig. 1). The treatment session lasted one hour. Every patient received 10 treatment sessions, 5 a week, for 2 weeks.

In parallel to the treatment sessions with the robotic hand, experimental group patients followed the usual daily rehabilitation treatment for subacute stroke inpatients at the “Clinica di Neuroriabilitazione, Ospedale Policlinico San Martino, Genova”. This rehabilitation program, tailored on the patient’s functional condition and lasting at least two hours a day, is based on passive, active-assisted and active exercises, focused on both the upper and lower limbs, but it does not include active or active-assisted exercises with the affected hand. Patients in the control group received this treatment only.

### Outcome measures

The compliance of patients was assessed in terms of active participation using the PRPS (primary outcome), which is a reliable and valid measure of inpatients’ participation in rehabilitation sessions. Participation is scored from 1 (refusal or no participation in any session) to 6 (excellent participation). We adopted PRPS because it has been shown a reliable and valid measure of inpatients’ participation in physical therapy, which can be used in clinical and research settings^21^.

The MAS was used to rate muscle tone of wrist and fingers flexor muscles (secondary outcome measure). The MAS rates muscle tone from 0 (no increased muscle tone) to 4 (rigid flexion or extension)^36^.

The motor section of the Fugl–Meyer Assessment Upper Extremity (FMA-UE) was used to assess the motor function of the upper limb. The motor section of FMA-UE evaluates aspects of movement, reflex, coordination, and speed. Each domain contains multiple items, each scored on a 3-point ordinal scale (0 = cannot perform, 1 = performs partially, 2 = performs fully). The motor section of FMA-UE is scored out of 66, with sub-scores of 24 for the wrist and hand^22^.

The Medical Research Council (MRC) scale for strength was used to rate muscle strength of wrist and fingers flexor and extensor muscles (0 = no contraction; 5 = normal strength).

### EMG assessment and measurement

Surface EMG from flexor muscles of the wrist and fingers was used to assess spasticity and spastic dystonia (secondary outcome measure). Surface pre-amplified electrodes with fixed inter-electrode distance of 20 mm (TSD150B, Biopac Systems Inc, USA) were placed over the muscle belly of Flexor Carpi Radialis (FCR) and Flexor Digitorum Superficialis (FDS) of the affected side, according to SENIAM guidelines^37^. Wrist joint angles were monitored by an electronic goniometer placed across the wrist joint (TSD130B, Biopac Systems Inc, USA). All signals were acquired by a Biopac MP100 unit (Biopac Systems Inc, USA) with a 2-kHz sampling rate. A Blackman-61 dB 10–350 Hz band-pass filter was used for offline processing (AcqKnowledge 3.8.1 software; Biopac Systems Inc, USA).

Subjects were seated in a chair with their back supported. For the entire duration of the recording session, subjects were instructed to stay completely relaxed and in silence. In each recording session, 5 EMG trials were collected. Each EMG trial included the following 3 subsequent phases:

- Phase 1 (looking for spontaneous tonic muscle activation): subject’s arms were arranged over his/her lap in the most natural position; EMG signal was recorded for approximately 10 s (s)
- Phase 2 (assessment of dynamic stretch reflex): the examiner (CT, medical doctor) grasped the subject's fingers and moved them and the wrist from the natural position of phase 1 to maximal extension in 1 s.
- Phase 3 (assessment of static stretch reflex): after the dynamic phase of the stretch, the subject's wrist and fingers were kept in the extended position for approximately 10 s.

To control the duration of the passive displacement, a method developed in our laboratory was used^38^.

The times corresponding to stretch onset and termination were visually detected on the goniometer trace displayed on the computer screen, using a display gain of 20°/cm and a temporal window of 340 ms/cm. The Average Rectified Values (ARVs) of spontaneous tonic muscle activation, dynamic stretch reflex and static stretch reflex were calculated using the dedicated AcqKnowledge analysis software (Biopac Systems).

When stretch reflex—but not spontaneous tonic muscle activation—was present, the hypertonic muscle was considered affected by spasticity. When both spontaneous tonic muscle activation and stretch reflex were present, the hypertonic muscle was considered affected by spastic dystonia.

### Time course

In patients of the experimental group, clinical assessments (except for PRPS) and EMG assessments were performed at the enrolment (before the first session of treatment) (T0), at the end of the last session of treatment (T1), 25 days after T0 (T2) and one year after stroke (T3). PRPS was performed after each therapy session.

In patients of the control group, FMA-UE and MAS scores were collected at the enrolment (T0) and 25 days after (T2).

### Statistical analysis

Descriptive statistics were reported as mean ± standard deviation. The Mann–Whitney U test was used to compare, both at T0 and T2, the FMA-UE and MAS scores between experimental group and control group. All statistical analyses were performed using Statview v5 and p-values < 0.05 were considered statistically significant.

## Supplementary Information

Supplementary Information.

## Data Availability

All data are available in the main text or the supplementary materials.
