# Supplementary material for: A soft supernumerary hand for rehabilitation in sub-acute stroke: a pilot study
Source: Sci Rep. 2022 Dec 13;12:21504. doi: 10.1038/s41598-022-25029-0 (PMC9747903; doi:10.1038/s41598-022-25029-0)
Supplement: Supplementary file 1 — Supplementary Information. [file 41598_2022_25029_MOESM1_ESM.docx]

Supplementary Materials for

A soft supernumerary hand for rehabilitation in sub-acute stroke: a pilot study

Carlo Trompetto, Manuel G. Catalano, Alessandro Farina,

Giorgio Grioli, Laura Mori, Andrea Ciullo, Matteo Pittaluga,

Martina Rossero, Luca Puce, Antonio Bicchi.

Correspondence to: giorgio.grioli@iit.it

**This PDF file includes:**

Table S1

**Other Supplementary Materials for this manuscript include the following:**

N/A

Table S1.

Clinical scores of the 4 patients, numeric values use to plot Fig. 2. Acronyms: FMA-UE (Fugl-Meyer Assessment for Upper Extremity), MAS (Modified Ashworth Scale), MRC (Medical Research Council scale for strength).

|  |  | **FMA-UE** | | **MAS** | | **MRC** | | | |
| --- | --- | --- | --- | --- | --- | --- | --- | --- | --- |
|  | **time** | **motor**  **function** | **hand-wrist**  **sub-score** | **wrist**  **flexors** | **finger**  **flexors** | **wrist**  **flexors** | **wrist extensors** | **finger**  **flexors** | **finger**  **extensors** |
| **Patient 1** | **T0** | 16 | 3 | **1** | **1** | 2 | 2 | 2 | 0 |
|  | **T1** | 28 | 8 | 0 | 0 | 3 | 3 | 3 | 2 |
|  | **T2** | 39 | 11 | 0 | 0 | 3 | 3 | 3 | 3 |
|  | **T3** | 65 | 24 | 0 | 0 | 5 | 5 | 5 | 5 |
| **Patient 2** | **T0** | 7 | 1 | **1** | 0 | 2 | 2 | 2 | 0 |
|  | **T1** | 23 | 8 | 0 | 0 | 3 | 3 | 3 | 2 |
|  | **T2** | 31 | 9 | 0 | 0 | 3 | 3 | 3 | 3 |
|  | **T3** | 62 | 24 | 0 | 0 | 5 | 5 | 4 | 5 |
| **Patient 3** | **T0** | 24 | 2 | **1** | 0 | 2 | 1 | 2 | 2 |
|  | **T1** | 34 | 7 | 0 | 0 | 3 | 2 | 3 | 3 |
|  | **T2** | 40 | 10 | 0 | 0 | 3 | 3 | 3 | 3 |
|  | **T3** | 59 | 21 | **1** | 0 | 5 | 5 | 5 | 5 |
| **Patient 4** | **T0** | 38 | 2 | **1** | **1** | 2 | 2 | 2 | 2 |
|  | **T1** | 44 | 8 | 0 | 0 | 2 | 3 | 3 | 3 |
|  | **T2** | 47 | 10 | 0 | 0 | 3 | 3 | 4 | 3 |
|  | **T3** | 64 | 24 | 0 | 0 | 5 | 5 | 5 | 5 |
